# Impaired integrity of commissural and association fibers in essential tremor patients:Evidence from a diffusion tensor imaging study

**DOI:** 10.3906/sag-2004-305

**Published:** 2021-02-26

**Authors:** Aygül TANTİK PAK, Yıldızhan ŞENGÜL, Hafize OTCU, Alpay ALKAN

**Affiliations:** 1 Department of Neurology, University of Health Sciences, Gaziosmanpaşa Research and Training Hospital, İstanbul Turkey; 2 Department of Neurology, Bezmiâlem Foundation University Hospital, İstanbul Turkey; 3 Department of Radiology, Bezmiâlem Foundation University Hospital, İstanbul Turkey

**Keywords:** Essential tremor, white matter, neurodegeneration, diffusion tensor imaging, fractional anisotropy, average diffusion coefficient

## Abstract

**Background/aim:**

The evolving understanding of essential tremors (ET) has led to a new definition of neurodegenerative disease, pointing to diffuse brain network involvement with a wide spectrum of associated motor and nonmotor symptoms. Considering the fact that white matter should also be affected by the nature of the disease, our study aimed to evaluate the integrity of white matter and its clinical correlations in ET patients.

**Materials and methods:**

Approximately 40 patients diagnosed with ET and 40 age-and sex-matched control subjects (ranging between 18–80 years old) were included in the study. The sociodemographic characteristics and clinical features of the patients were recorded. Tremors were assessed using the Fahn-Tolosa-Marin Tremor Rating Scale (FTM-TRS). Diffusion Tensor Imaging (DTI) was performed to evaluate the integrity of white matter. The selected white matter regions used for DTI assessment were the corpus callosum (CC) (i.e., the largest commissural tract in the human brain), the superior longitudinal fasciculus (SLF), and the inferior longitudinal fasciculus (ILF) (i.e., the largest association fiber bundles).

**Results:**

The mean age of the ET patients and control subjects was 44.23 ± 18.91 and 37.45 ± 10.95 years old (P = 0.542). The fractional anisotropy (FA) values of the CC body (P = 0.003), ILF (p = 0.016), average diffusion coefficient (ADC) values of the CC body (p = 0.001), genu (P = 0.049), SLF (V < 0.001), and ILF (P < 0.001) differed between groups. After controlling for age and sex, there was no correlation between tremor severity and DTI parameters, but impaired integrity in the genu of CC FA (P = 0.035, r = 0.442) and the splenium of CC ADC (P = 0.007, r = 0.543) were related with a longer duration of tremor. Finally, positive family history was correlated with the splenium of CC FA and ADC (P = 0.008, r = 0.536; P = 0.027, r = 0.461) and ILF ADC (P = 0.011, r = –0.519).

**Conclusion:**

In our study, major white matter structure changes were found in the ET patients. The results suggest that possible neurodegeneration also affects white matter structures in ET patients and that the duration of the tremor and family history are related with impaired integrity of white matter.

## 1. Introduction

An essential tremor (ET) is one of the most common movement disorders globally [1,2]. The prevalence of ET is 4.0% for ages 40 and above [3]. It is a slowly progressive disorder characterized by postural and kinetic tremors occurring predominantly in the forearms and hands, ultimately spreading to the head and other body regions [1,4]. ET is also associated with a number of motor and nonmotor manifestations, including cognitive deﬁcits, neuropsychiatric symptoms [anxiety, depression, speciﬁc personality traits), sleep disorders, and sensory deficits [5–16]. In recent years, according to systematic postmortem and neuroimaging studies, our knowledge of the symptomatology and neuropathology of this disease has grown substantially. Postmortem examinations in ET patients revealed reactive gliosis in the cerebellum, depletion of locus coeruleus neurons, and Lewy body pathology [17–19]. Early changes in the cerebellar cortex include damage in both the axonal and dendritic purkinje cell compartments, which eventually leads to purkinje cell death. As a result, reduced purkinje cell inhibitory output and impaired fiber connections between the cerebellar cortex and the dentate nucleus have been observed [20,21]. Functional and structural neuroimaging studies have identified the involvement of cerebellar pathways [16,22–29]. 

Neuroimaging studies have also demonstrated structural changes in grey matter regions as frontal, temporal, and occipital cortex [22,30–33], insula, precuneus [22,31], basal ganglia, red nucleus, substantia nigra, and thalamus [34]. The neurodegenerative nature of the disease and a number of accompanying nonmotor symptoms suggest that this neurodegeneration may also spread to white matter. Thus, several recent studies reported changes in white matter structures [34–37]. In addition to the impaired integrity of cerebellar peduncles [36], researchers reported disease-related changes in the corticospinal tract, internal capsule, superior longitudinal fasciculus, (SLF) and corpus callosum (CC) in a limited number of ET studies [36–38].

Diffusion tensor imaging (DTI) is a magnetic resonance imaging (MRI) technique that is used to map the three-dimensional diffusion of water as a function of spatial location. Several DTI parameters are used to assess diffusion and, indirectly, fiber tract microstructure. Fractional anisotropy (FA) measures the anisotropic diffusion of water molecules, and the average diffusion coefficient (ADC) describes the magnitude of the average molecular displacement by diffusion. DTI shows where neuronal/axonal loss occurs as a result of neurodegeneration [39]. 

The aim of this study was: (i) to show possible white matter involvement in ET neuropathology using region of interest (ROI) DTI method and (ii) to evaluate the correlations of these possible white matter alterations.

## 2. Materials and methods 

### 2.1. Participants

In this observational case control study, we recruited 84 (ranging between 18–80 years old) consecutive patients with an initial diagnosis of ET between March 2018 to July 2018. The study was conducted according to the Helsinki Declaration’s ethical principles and was approved by the Bezmiâlem Foundation University Hospital Ethics Committee. Written informed consent was obtained from the participants after details of the procedures were fully explained. All participants were carefully examined by a movement disorder specialist. A diagnosis of ET confirmed by using the Movement Disorder Society criteria [40]. Our exclusion criteria were: (i) metabolic causes of tremor (vitamin B12 or folate deficiency, anemia, hypo or hyperthyroidism), (ii) causes of possible brain damage (e.g., history of significant head trauma, brain surgery or stroke history), (iii) mental disorders or dementia, (iv) a clinical dementia rating (CDR) [41] scale score >0.5 (all patients aged 65 years and older were administered CDR), (v) and use of medication known to cause tremors. Patients with prosthetics, metal inserts (metal knee caps and pacemakers) not compatible with MRI, together with claustrophobic patients, were excluded from the study. As a result of these exclusion criteria, the final sample number in our patient group (we initially started with 84 patients) was 40 patients. Therefore, 40 age and sex-matched control subjects were included in the study group among patients’ nonblood relatives and hospital workers.

### 2.2. Clinical evaluation

Sociodemographic characteristics, family history (first and second degree), disease duration, and tremor localizations of the patients were recorded. The patients were placed into 2 groups according to their tremor localizations. Group 1 consisted of patients with a tremor only on upper extremities; Group 2 consisted of patients with a cranial tremor accompanying upper extremities. The Fahn-Tolosa-Marin Tremor Rating Scale (FTM-TRS) was used to evaluate tremor severity [42]. This scale is a method for measuring resting, postural, and action tremors. There are 5 scores (0–4) for each category that represent severity levels. Increasing scores mean increasing severity of the disease. 

### 2.3. MRI protocol

All patients were examined using a 1.5T MRI system (Siemens, Avanto, Erlangen, Germany) with a maximum gradient strength of 43 mT/m and an 18-channel head coil. First, routine brain imaging protocol included T1-weighted (T1W) spin echo (TR/TE, 460/14 ms), T1W with fat suppression (TR/TE, 715/7.5 ms) without contrast, T2-weighted (T2W) turbo spin echo (TR/TE, 2500/80 ms), and FLAIR (TR/TE, 8000/90 ms) sequences. The 3D T1W volumetric sequences (TR/TE/TI, 12.5/5/450 ms) without contrast were then applied using a magnetization-prepared rapid acquisition of gradient echo sequence (MPRAGE) with an isotropic voxel resolution of 1 mm. Parallel imaging using generalized autocalibrating partially parallel acquisition (GRAPPA) with an integrated parallel acquisition technique (iPAT) factor of 2 applied. The DTI protocol included a single-shot, spin-echo, echo-planar sequence with TR/TE, 2700/89 ms; matrix, 128 × 128; field of view, 230 mm; a slice thickness of 5-mm and 64 diffusion-encoding directions were used at b = 0 s/mm2 and b = 1000 s/mm2. Parallel imaging using GRAPPA with an iPAT factor of 3 was applied. A semiautomated system was used for ROI-based DTI analysis. The ADC and FA maps were reconstructed on a Leonardo console (version 2.0, Siemens) utilizing the DTI data. In addition, 3D T1 and T2-weighted images were used as anatomic references at the placement of the ROIs. These images were then matched with the corresponding regions in the ADC and FA maps at the same anatomic level. The ROIs were drawn manually on axial color-encoded FA maps in all subjects with simultaneous assessment of an experienced radiologist (HO) who identified the adaptation of the sizes and placement of the ROIs by dissecting white matter tracts with an interactive DTI teaching atlas [43]. Localization regions were CC (genu, body, splenium) (i.e. the largest commissural tract), SFL, and inferior longitudinal fasciculus (ILF) (i.e. the largest bundle of association fibers). To standardize the measurements, all ROIs were obtained from the left side.

### 2.3. Statistical analysis 

All statistical analyses were performed using a commercially available SPSS release 20.0 software package (IBM Corp., Armonk, NY, USA). The results were presented as mean ± standard deviation. A Chi-square test was used to compare groups. The Kolmogorov–Smirnov test was used to assess normality. According to the distribution of the data, an independent t-test or Mann–Whitney U test was used to compare groups. Partial correlation was used to evaluate all correlations between the duration of the tremor, FTM-TRS scores, family history, and DTI metrics controlling for age and sex. P < 0.05 was accepted as statistically significant.

## 3. Results

The mean age of the ET patients (n = 40) was 44.23 ± 18.91 years (ranging between 18–71 years); 70% of the patients were female (n = 28) and 30% were male (n = 12). The mean score of the FTM-TRS scale was 21.03 ± 9.12 for all patients. The mean duration of tremors was 10.60 ± 8.86 years. Positive family history was found in 70% of the patients (n = 28). The percentage of patients with a tremor strictly localized in the hands was 82.5% (n = 33), and the percentage of those with a tremor in other body regions accompanying the hands was 17.5% (n = 7) (Table 1). 

**Table 1:** Sociodemographic of participants and clinical characteristics of ET patients.

	ET patients(n = 40)	Healthy controls(n = 40)	P* value
Age	44.23 ± 18.91	37,45 ± 10,95	0.54
Sex: FemaleMale	%70 (n = 28)%30 (n = 12)	%50 (n = 20)%50 (n = 20)	0.11
Clinical characteristics of ET patients			
Duration of tremor (years)	10.60 ± 8.86		
Mean score of FTM-TRS***	21.03 ± 9.12		
Family historyPositive:Negative:	70 % (n = 28)30 % (n = 12)		
Tremor localizationUpper extremities:Other body regions:	82.5 % (n = 33)17.5% (n = 7)		

*Chi-square test; ET: essential tremor; FTM-TRS: Fahn-Tolosa-Marin Tremor Rating Scale.

The study group was divided into 2: those with ET (n = 40) and the control group (n = 40). There were no significant differences between the groups when comparing age (44.23 ± 18.91 vs. 37.45 ± 10.95 years, P = 0.542) and sex (70% for female vs. 50% for male, P = 0.110) (Table 1).

Comparison of major white matter tracts between groups showed significant changes in the ET group in CC body FA and ADC (P = 0.003, P = 0.001), CC genu ADC (P = 0.049), SLF ADC (P < 0.001), and ILF FA and ADC (P = 0.016, P < 0.001) (Table 2). 

**Table 2:** Comparison of DTI metrics between groups (FA and ADC values are presented in units of 10−6 mm2/s).

		ET patients(n = 40)	Healthy controls(n = 40)	P value
Superior longitudinal fasciculus	FA	530.87 ± 68.04	554.97 ± 77.12	0.126*
	ADC	725.67 ± 43.58	771.85 ± 44.26	< 0.001*
Inferiorlongitudinal fasciculus	FA	543.77±96.64	496.80 ± 72.44	0.016**
	ADC	843.70 ± 83.95	752.85 ± 60.58	< 0.001*
Corpus callosum Splenium	FA	795.92 ± 83.96	788.17 ± 50.84	0.223*
	ADC	817.82 ± 78.76	811.90 ± 59.91	0.908*
Corpus callosum body	FA	625.67 ± 84.62	681.12 ± 80.96	0.003*
	ADC	1098.10 ± 294.82	944.40 ± 204.25	0.001*
Corpus callosum genu	FA	781.72 ± 64.20	802.35 ± 55.38	0.128**
	ADC	851.07 ± 103.33	812.70 ± 78.76	0.049*

*Independent sample t-test; **Mann–Whitney U Test; Bonferroni corrected p-value is 0.01; FA: fractional anisotropy; ADC: average diffusion coefficient.

The evaluation of partial correlation controlling for age and sex between family history and DTI data resulted in a significant correlation between the splenium of CC FA and ADC ADC (P = 0.008, r = 0.536; P = 0.027, r = –0.461) and ILF ADC (P = 0.011, r = –0.519). No significant association was found between the FTM-TRS scale and DTI data (P > 0.05). Tremor duration and the genu of CC FA value (P = 0.035, r = 0.442) and the splenium of CC ADC value (P = 0.007, r = 0.543) showed a significant association. There was no significant relationship between tremor localization and DTI parameters (Table 3).

**Table 3:** Evaluation of partial correlation controlling for age and sex between clinical data in ET patients and DTI metrics.

		Family history*		FTM-TRS*		Tremor duration*		Tremor localization*	
		r value	P value	r value	P value	r value	P value	r value	P value
Superior longitudinal fasciculus	FA	0.394	0.063	-0.299	0.166	0.082	0.710	-0.123	0.577
	ADC	-0.180	0.412	0.287	0.184	0.203	0.353	0.176	0.421
Inferior longitudinal fasciculus	FA	0.235	0280	0.062	0.777	0.076	0.730	0.075	0.733
	ADC	-0.519	0.011	-0.096	0.663	0.259	0.233	-0.137	0.534
Corpus callosumgenu	FA	0.536	0.008	-0.404	0.056	-0.442	0.035	0.011	0.961
	ADC	-0.461	0.027	0.130	0.554	0.221	0.312	0.113	
Corpus callosumbody	FA	0.162	0.459	0.048	0.827	-0.009	0.968	-0.226	0.301
	ADC	0.069	0.753	*0.270	0.213	-0.216	0.322	-0.029	0.896
Corpus callosum splenium	FA	0.109	0.621	-0.299	0.166	-0.330	0.125	0.190	0.385
	ADC	-0.044	0.842	-0.111	0.615	0.543	0.007	-0.295	0.173

*Partial correlation analysis controlling for age and sex; FTM-TRS: Fahn-Taloso-Marin Tremor Rating Scale.

## 4. Discussion

It is essential to identify the affected brain regions in ET in order to understand this disease more comprehensively. Our study was planned considering the fact that the integrity of major white matter bundles (CC, SFL, and inferior longitudinal fasciculus) might be impaired and that this finding could reflect the neuropathology of the disease. As a result of this study, microstructural tissue breakdown was found in all 3 major white matter regions. Microstructural changes of the splenium of CC and ILF were positively correlated with family history. In addition, microstructural changes in the genu of CC and the splenium of CC were related to longer duration of tremors. 

As we mentioned above, there have been several studies conducted with small sample sizes on this subject. In a study comparing the DTI results of 10 ET patients and 8 control groups, a significant decrease in the FA value was detected in the right half of the pons, bilateral cerebellum, left retrobulbar area and orbitofrontal, lateral frontal, parietal, and temporal deep white matter [27]. In another study that assessed white matter abnormalities in ET for 14 ET patients and 20 control group participants, both ROI-based model and tract-based model DTI methods were used. Using ROI analysis, increased MD bilaterally in the inferior cerebellar peduncles and reduced FA in the right-sided inferior cerebellar peduncles, as well as increased FA in left parietal white matter of ET patients, were all detected. As a result, microstructural alterations in the cerebellum and cerebellar white matter were identified [36]. 

In Saini et al.’s study, clinical and MRI data from 20 patients with ET and 17 controls were collected prospectively. The DTI data were analyzed using tract-based spatial statistics (TBSS). Further ROI analysis was carried out in the genu of CC, internal capsule, corticospinal tract, and cerebellar peduncles. Patients with ET, in comparison to controls, showed a significant increase of mean diffusivity (MD) in the right internal capsule and left corticospinal. An axial diffusivity (AD) increase was seen in the right inferior cerebellar peduncle and corticospinal tract. ROI analysis also revealed a decrease in FA values in the left superior cerebellar peduncle and right corticospinal tract. As can be seen, the cerebellum and associated pathways have been shown to be affected in most of the studies by large white matter bundles such as the CC [37].

Nestrasil et al. investigated the correlation between tremor severity and neuroimaging in ET patients. Tract-based and voxel-based approaches were utilized to compare DTI data from 12 ET patients and 10 age-and sex-matched healthy individuals. The ET patients demonstrated significant correlations between bilateral corticospinal tracts, superior longitudinal fascicles, and the CC but also in nonmotor regions, including the inferior fronto-occipital and longitudinal fascicles, cingulum bundles, anterior thalamic radiations, and uncinate fascicles. The results show significant correlations between objective tremor measures (combined TremScore, tremor frequency) and diffusivity metrics, primarily in the white matter regions. In contrast, a relationship between white matter changes and FTM-TRS was not detected in the present study [38]. These conflicting results on the relationship between tremor severity and DTI parameters could be the result of the larger sample size used in our study. In addition to FTM-TRS, Nestrasil et al. used accelerometry to evaluate tremor severity. Accelerometry tremor parameters showed robust correlation with changes in the white matter of the primary and associative motor areas, which were not detectable with FTM-TRS. The lower interrater reliability and subjective nature of the FTM-TRS could also explain the different results detected in our study. 

Recently, a neuroimaging study investigating the further deterioration of white matter compared to grey matter deterioration of brain structures conducted with 19 ET patients and 15 control group individuals. White matter abnormalities were detected in the corticospinal tract, anterior thalamic radiation, SFL and inferior longitudinal fasciculus, inferior fronto-occipital fasciculus, and superior and middle cerebellar peduncles in ET patients compared to the control group. White matter abnormalities (CC, corticospinal tracts, SLF, and cerebellar peduncles) significantly correlated with tremor severity [44]. This study demonstrated that while the grey matter damage in ET was limited to the thalamus, white matter damage was widespread, involving the majority of white matter bundles. In our study, similarly, significant DTI differences were observed between ET patients and CS in major white matter bundles. Again, in contrast with this study, we did not find a relationship between the DTI parameters and tremor severity, except in terms of the duration of the tremor.

When interpreting our DTI results, one should consider that many things can affect FA (and the other diffusion metrics such as ADC, MD, radial diffusivity [RD], and AD). In addition, while FA values typically decrease in disease and in the presence of neurodegeneration, neuroinflammation sometimes can have the opposite effect on FA, and this is similar in other values. For example, acute shear injury can produce axonal retraction balls thought to increase FA or, in acute stroke, FA is increased through a relatively greater decrease in the isotropic diffusion component. Considering RD and AD, one should consider that the exact pathophysiological representation of these changes is still an active area of research [45–49]. 

This study must be interpreted considering several limitations: only an ROI-based model was used as the DTI method, and a tract-based model was not used. Also, specific white matter structures were evaluated in our study; however, whole-brain analysis of the data was not performed.

## 4. Conclusion

Our results showed impaired integrity of major white matter bundles in the brains of ET patients, which supports the hypothesis of possible widespread neurodegeneration in ET involving white matter structures. Previously, there have been a limited number of DTI studies in ET confirming involvement of brain white matter structures. Similar findings were also observed in our study. However, the correlation of white matter microstructural changes with motor and nonmotor signs in ET remains unclear. Evaluation of the relationship between both motor and nonmotor symptoms and white matter structures in future studies can provide information that will lead to a better understanding. 

## Informed consent

The study was conducted according to the Helsinki Declaration’s ethical principles and was approved by the Bezmiâlem Foundation University Hospital Ethics Committee. Written informed consent was obtained from the participants after the details of the procedures were fully explained.
